# A facile hydrothermal approach for catalytic and optical behavior of tin oxide- graphene (SnO_2_/G) nanocomposite

**DOI:** 10.1371/journal.pone.0202694

**Published:** 2018-10-01

**Authors:** Suresh Sagadevan, Zaira Zaman Chowdhury, Mohd. Rafie Bin Johan, Ali. A. Khan, Fauziah Abdul Aziz, Rahman F. Rafique, Md Enamul Hoque

**Affiliations:** 1 Centre for Nanotechnology, AMET University, Chennai, India; 2 Department of Physics, Center for Defence Foundation Studies, National Defence University of Malaysia, Kem Sungai Besi, Kuala Lumpur, Malaysia; 3 Nanotechnology & Catalysis Research Centre, University of Malaya, Kuala Lumpur, Malaysia; 4 Rutgers Cooperative Extension Water Resources Program, Rutgers, The State University of New Jersey, New Brunswick, New Jersey, United States of America; 5 Department of Biomedical Engineering, Military Institute of Science and Technology (MIST) Mirpur Cantonment, Dhaka, Bangladesh; Institute of Materials Science, GERMANY

## Abstract

A cost-effective, facile hydrothermal approach was made for the synthesis of SnO_2_/graphene (Gr) nano-composites. XRD diffraction spectra clearly confirmed the presence of tetragonal crystal system of SnO_2_ which was maintaining its structure in both pure and composite materials’ matrix. The stretching and bending vibrations of the functional groups were analyzed using FTIR analysis. FESEM images illustrated the surface morphology and the texture of the synthesized sample. HRTEM images confirmed the deposition of SnO_2_ nanoparticles over the surface of graphene nano-sheets. Raman Spectroscopic analysis was carried out to confirm the in-plane blending of SnO_2_ and graphene inside the composite matrix. The photocatalytic performance of the synthesized sample under UV irradiation using methylene blue dye was observed. Incorporation of grapheme into the SnO_2_ sample had increased the photocatalytic activity compared with the pure SnO_2_ sample. The electrochemical property of the synthesized sample was evaluated.

## 1.Introduction

Recently research interest revolved around the fabrication of semiconductor metal oxide nanoparticles with tunable morphologies and distinct properties. Based on their structure and synthesis strategy, the electrical, optical and catalytic properties can be altered significantly. Tin-oxide (SnO_2_) with different architectural structure including 1D nanorods, 2D nanosheets and 3D nanostructure can exhibit fast charge transportation properties [1] which enable them to be used as promising candidate for electrode preparation. To date, the reported synthesis process of tin-oxide nanoparticles need multiple steps with manipulation of process variables which is less efficient and time consuming [2, 3].The literature has reported on growing SnO_2_ nanorods onto the surface of graphene nanosheets that require some organic compounds for their synthesis process. During the synthesis process, these compounds strongly adsorbed over the surface of SnO_2_ and the surface active sites are blocked resulting in poor performance of the system [4, 5]. Graphene-a 2D nano carbon can provide extra-large surface area which enables them to fabricate hybrid electrode materials with high performance [6–8]. It has attracted a lot of attention of researchers due to its excellent optical [9–11], mechanical [12, 13] and electrochemical properties [14–17]. In addition, SnO_2_ exhibits good photocatalytic properties for degrading dyes under UV light, particularly for SnO_2_ nanocomposites due to electron transfer between SnO_2_ and an additive [18]. Graphene is a good choice as an additive because it is an exciting two-dimensional material with a large specific surface area and perfect electrical properties [19]. Li et al., deposited SnO_2_ on graphene sheets by reducing graphite oxide with SnCl_2_ in the presence of hydrochloric acid and urea. The resulting nanocomposite showed good capacitive properties [20]. Kim et al., reported a straightforward method for fabricating graphene-SnO_2_ composites with high lithium storage capability by adding NH_4_OH and hydrazine to RGO followed by the oxidation of tin particles with HCl [21]. Most of the research works concentrated on improving various electrode materials such as conducting polymers, metal oxides out of which carbon based materials such as activated carbon, carbon nanotubes and carbon aerogels are given more consideration for energy storage devices [22–36]. Recently research interest has been rapidly growing for the fabrication of semiconductor metal oxide nanoparticles with tunable morphologies and distinct characterization. Based on their structure and synthesis strategy, the electrical, optical and catalytic properties can be altered significantly [37–42].

Herein, we synthesized SnO_2_/G composites using a facile, cost-effective hydrothermal method whereby all the reaction parameters were carefully optimized to retain the crystalline structure of SnO_2_ similar to its bare metal oxide. The synthesized composite was used to study the photocatalytic degradation of MO and MB under visible light irradiation. The electrochemical properties were analyzed to observe the synergistic effect of graphene and SnO_2_ nanomaterials. The electrosorption desalination performance of the synthesized sample was observed. The results showed that the synthesized sample might have a good potential to be used as electrode materials for super capacitor application having high energy.

## 2.Experimental

### 2.1.Material

Natural Graphite flakes, Stannous chloride pentahydrate (98%, SnCl_4_.5H_2_O), Sodium hydroxide (NaOH), potassium hydroxide (KOH) and ethanol which was used here were of analytical grade. Thus the chemicals obtained were used without any purification.

### 2.2.Preparation of SnO_2_/G nanocomposite

Graphene oxide (GO) was prepared by modified Hummers method. The SnO_2_/G nanocomposite was synthesized by adding SnCl_4_.5H_2_O (0.1 M) in 40 ml of uniformly dispersed GO solution (1mg/ ml) and NaOH was added to adjust the pH up to 9. The prepared mixture was kept in sealed Autoclave at 180°C for 4 hrs which yielded a composite black in colour. The product was further washed by centrifugation in ethanol and water several times at 5000 rpm for maximum purification. The end product was calcined at 300°C for 12 hrs.

### 2.3.Electrode preparation

The working electrode was prepared by mixing the electro active material of SnO_2_/G (80 wt %), activated carbon (10 wt %), and polyvinylidene fluoride (10 wt %). Finally, the acquired slurry was coated onto nickel foil (1 cm^2^) and dried at 100°C in air for 12 h. Every electrode included nearly 1 mg of electro active material. The SnO_2_/G electrode as the working electrode, Pt wire as counter electrode and the saturated calomel electrode as reference were employed for further study. Aqueous 2 M KOH solution was used as the supporting electrolyte. CVs in the potential range of -0.4 to 1 V at various current densities and electrochemical impedance spectra (EIS) of amplitude were carried out by using VSP biologic electrochemical system.

## 3. Results and discussion

**Fig 1:**
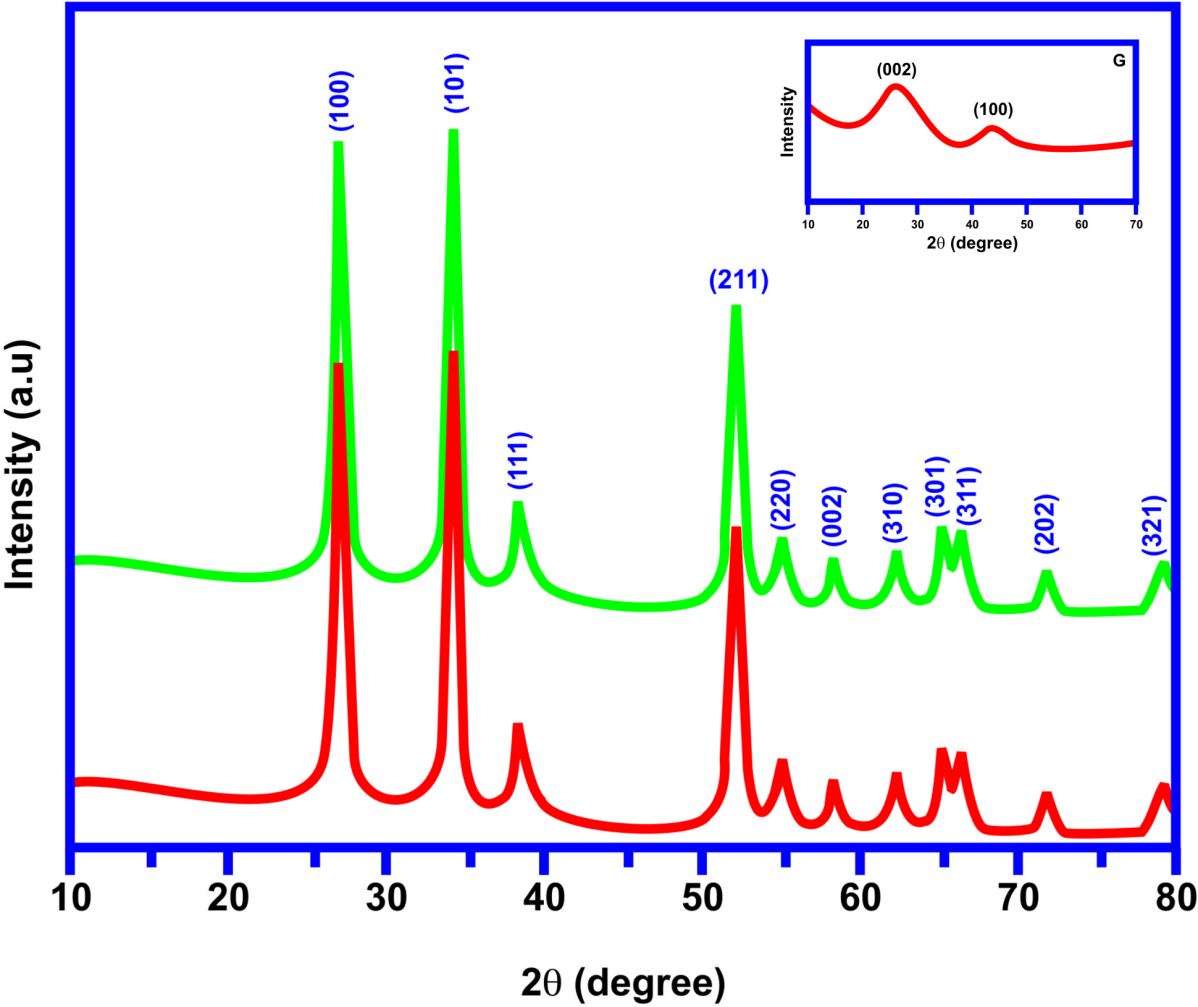
X-ray diffraction pattern of SnO_2_/G nanocomposite.

Fig 1 illustrates the XRD pattern of the synthesized SnO_2_/G nano-composite. The tetragonal rutile structure of SnO_2_ inside the composite matrix was confirmed by the XRD diffraction pattern. The Graphene XRD pattern is shown in the insert Fig 1. The good crystallinity of SnO_2_ particles were confirmed by the strong intensity of the peak. The diffraction peaks of SnO_2_/G nanocomposite were indexed with (100), (101), (111), (211), (220), (002), (310), (301), (311), (202), and (321) planes. There were no diffraction peaks because graphene could be identified in the composite. The vanishing of diffraction peaks of graphene layer may be due to the peeling of GO sheets during the hydrothermal process. Besides, the connected SnO_2_ nanoparticles on graphene sheets keep the aggregation and restacking of graphene and because of the excellent crystallinity of SnO_2_ in the composite, the diffraction of carbon atoms in graphene is depleted. In this manner, the SnO_2_ nanoparticles covering the graphene sheets provide strong diffraction in the composite.

**Fig 2:**
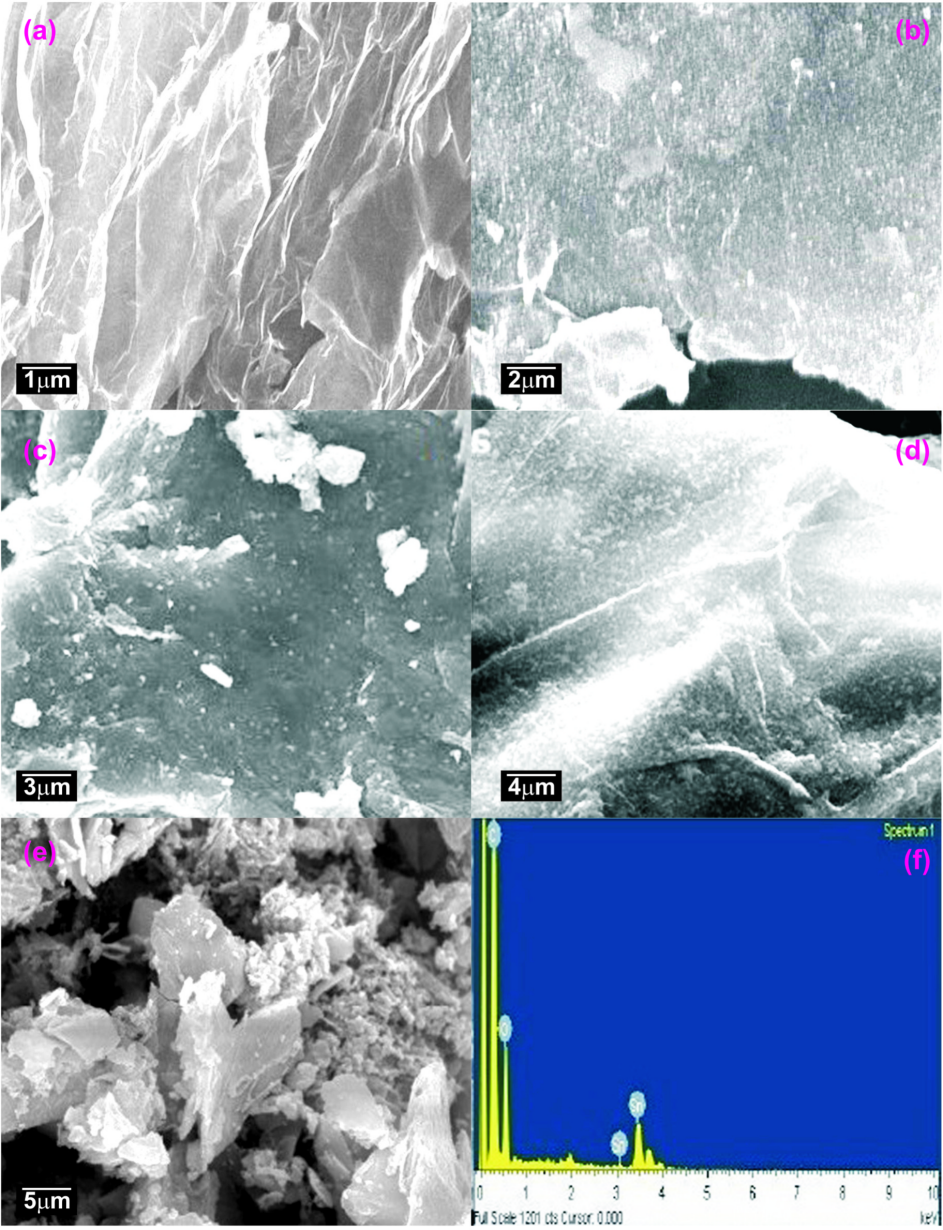
FE-SEM images of (a—e) SnO_2_/G nanocomposite and (f) EDX spectrum of SnO_2_/G nanocomposite.

**Fig 3:**
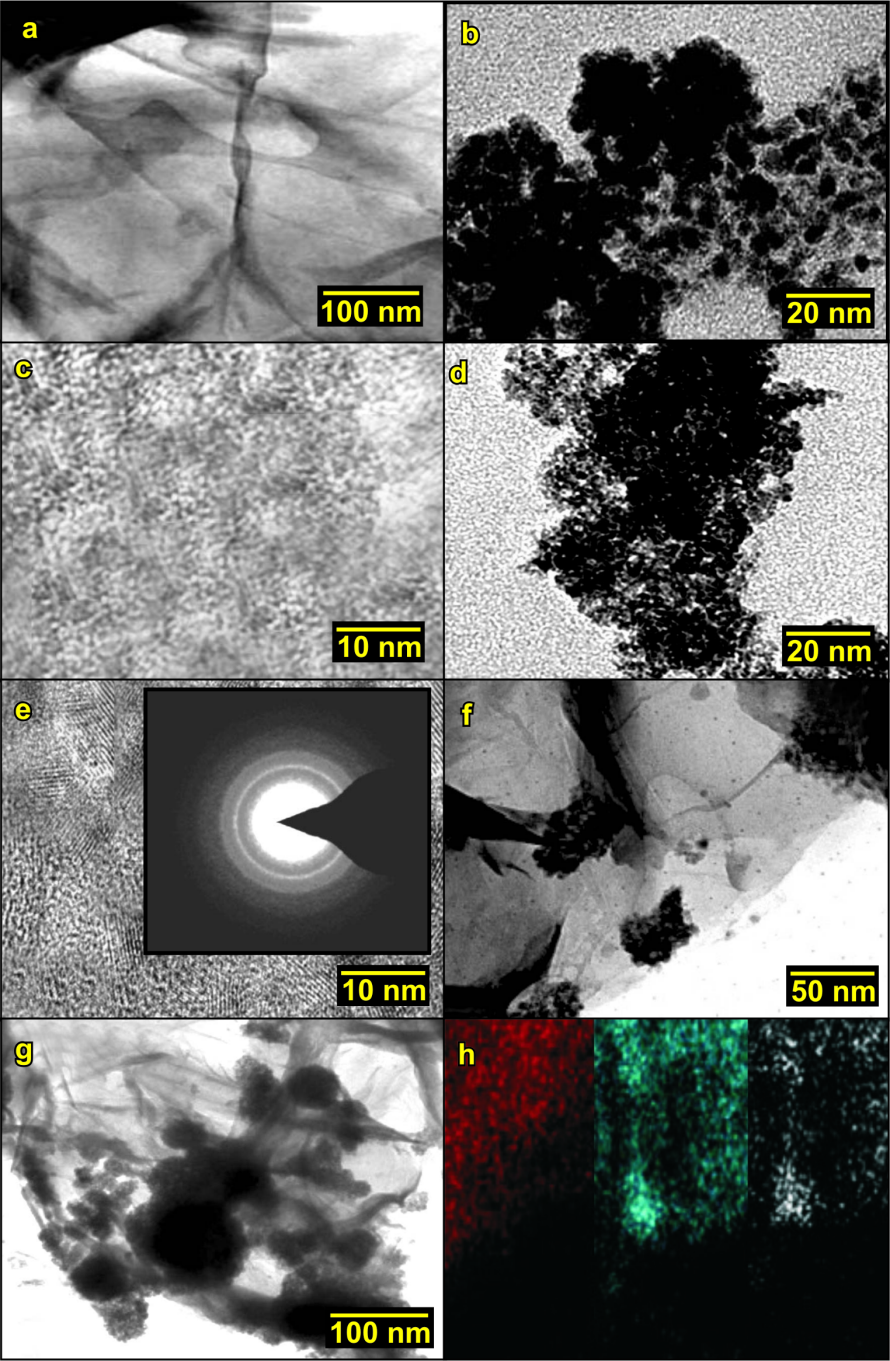
(a, b) TEM image of Graphene and SnO_2_ (c-g) TEM and HR-TEM images of SnO_2_/G nanocomposite and (f) Elemental mapping of SnO_2_/G nanocomposite.

The FE-SEM images of the synthesized samples are shown by (Fig 2A–2E).The SnO_2_/G nanocomposite (Fig 2A–2D) showed platelet like structure. It formed like a very thin sheet like structure. Nano-sized SnO_2_ particles were clearly visible over the surface of the graphene sheets. The sheet-like morphology was retained even after covering the surface of the graphene with an interconnected network of SnO_2_/G nanocomposite. (Fig 2A–2E) illustrates the homogeneous distribution of SnO_2_ nanoparticles with a narrow size distribution over the graphene sheets. This indicates strong interaction between SnO_2_ and graphene nanosheets. The restacking of graphene nano sheets was prevented by the presence of SnO_2_ nanoparticles. Consequently, it increased the stability of the individual graphene sheets and prevented the aggregation of the SnO_2_ nanoparticles. Fig 2(F) illustrates the EDS pattern of the SnO_2_/G nanocomposites where only C, O, and Sn atoms have been observed. The TEM and HR-TEM images of the Graphene, SnO_2_ and SnO_2_/G nanocomposites are shown in (Fig 3A–3G). (Fig 3A–3G) demonstrates the TEM and HR-TEM images of the synthesized sample. The homogeneous dispersion of SnO_2_ nanoparticles over as well as the interlayers of the graphene sheets has been observed from the HR-TEM image as shown in (Fig 3A–3E). The higher magnification image clearly confirms the presence of the layered structure of graphene and SnO_2_ nanoparticles as shown in (Fig 3C–3E). Fig 3(F) shows the elemental mapping of the SnO_2_/G Nano-composite. The homogeneous atomic distribution for C, O, and Sn were observed.

**Fig 4:**
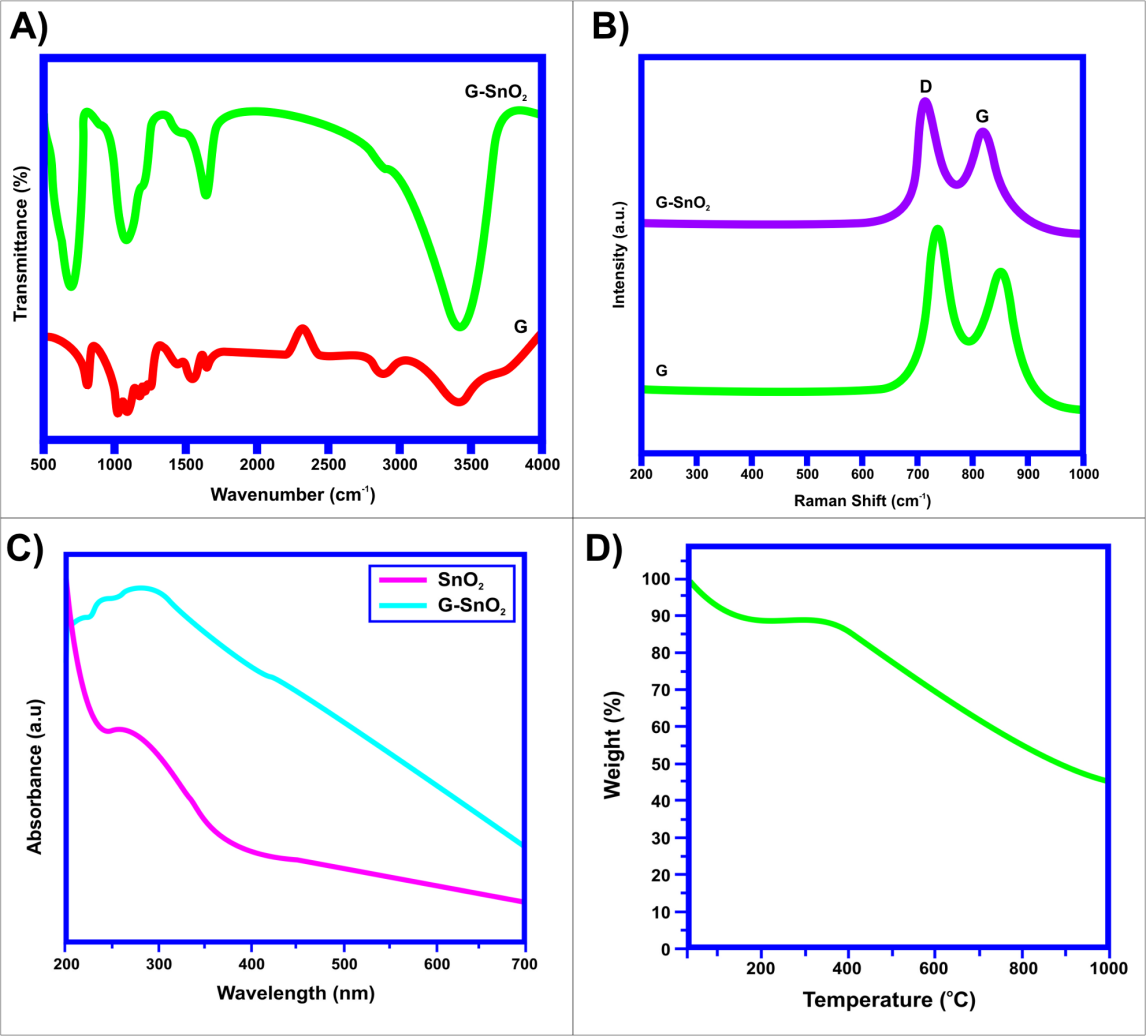
(a) FTIR Spectrum of SnO_2_/G Nanocomposite (b) Raman Spectrum of SnO_2_/G Nanocomposite (c) UV-Vis spectrum of SnO_2_/G nanocomposite (d) TGA curve of SnO_2_/G nanocomposite.

The surface functional groups over the surface of graphene and SnO_2_/G nanocomposites were observed using FTIR analysis and the same are shown in Fig 4(A). FTIR spectrum shows the SnO_2_/G composite, in which the absorption peak at 3435 cm^-1^ corresponds to the stretching vibration of OH. The characteristic peak observed at 1726 cm^-1^ was owing to the presence of C = O stretching vibration of carboxyl group. The presence of absorption peak around 1624 cm^-1^ could be ascribed to C = C aromatic ring vibration. The peak at 1214 cm^-1^ showed the presence of C-OH stretching bond over the surface of SnO_2_/G [43]. The small peak at 840 cm^-1^ could be attributed to O—C = O groups [44]. A peak around 1562 cm^-1^ represented the vibration of graphene sheets [45]. The peak around 562 cm^-1^ was assigned to Sn-OH vibration. The peak at 672 cm^-1^ was ascribed to O-Sn-O stretching vibration of tin-oxide. Both of these peaks confirmed the formation of SnO_2_ nanoparticles.

The Raman spectrum of the graphene and SnO_2_/G nanocomposite also confirmed the coating of SnO_2_ nanoparticles over the graphene sheets and it is illustrated in Fig 4(B). The D and G bands were observed at 1322 cm^-1^ and 1594 cm^-1^. These two peaks showed the vibrational modes of SnO_2_ nanoparticles. The strong peak in the range of 1580–1600 cm^-1^ for graphene showed the aromatic ring chain vibrations. The D band observed at 1352 cm^-1^ was ascribed to the plane imperfection related to the breathing mode of aromatic rings and k-point phonon of A_1g_. The G band that appeared at 1594 cm^-1^ was reflecting the plane vibration of an E_2g_ phonon of sp^2^ hybridization of carbon atoms [46]. Basically D band is stronger than G band. The magnitude of D/G ratio increased considerably compared with the pristine graphite sample. This phenomenon supported the presence of graphene nanosheets inside the composite materials. When SnO_2_ nanoparticles were placed inside the graphene nanosheets–there was a decrease in SP^2^ carbon species in graphene. Thus D/G ratio for SnO_2_/G sample was higher than for the pure graphene sample.

UV–visible (UV–Vis.) absorption spectroscopy is widely used as a primary means of assaying concentrations and in characterizing the kinetics of reactions and chemical processes; it is ubiquitously used for chromatographic detection. To confirm the tunability of the optical properties of the SnO2/G nanocomposite, UV absorption spectroscopy was used. While in optical UV–Vis. absorption spectrum of as synthesized SnO_2_/G nanocomposite was calculated at a wavelength ranging from 200 to 700 nm. The UV-Vis absorption spectrum of the synthesized nanocomposite is illustrated in Fig 4(C). For the graphene, red shift was observed in the range from 290 nm to 270 nm following the reduction. A broad absorption peak was observed with the absorption maximum at 285 nm for SnO_2_/G nanocomposites with a noticeable red shift in the absorption maximum. This red shift was attributed to the presence of semiconductor SnO_2_ nanoparticles on the graphene sheet. It is interesting to note that in the case of SnO_2_/G nanocomposites, the peak at 280 nm associated with graphene red-shifts to 285 nm gradually with increasing centrifugation rate, indicating an increase in the π-electron concentration with reduction of sp^3^ graphene oxide to sp^2^-hybridization of carbon atoms [47]. For a smaller size of nanocrystals, the self-assembly process results in a more efficient reduction of graphene due to possible charge transfer in SnO_2_/G nanocomposites.

Thermo Gravimetric analysis was carried out to analyze the thermal stability of graphene and graphene-tin oxide nanocomposite. The analysis was done at a heating rate of 10°C/min. The temperature range for that analysis was 30 ^o^C to 1000 ^o^C. A major weight loss starting from 180°C to 255°C was observed for G. This was due to the pyrolysis of the same functional groups which would form CO, CO_2_, and vapour. From Fig 4(D) it is evident that no appreciable weight loss was observed for the SnO_2_/G nanocomposite up to 1000°C. This demonstrates the high stability and purity of the SnO_2_/G nanocomposite. Extensive weight loss was observed between 500°C and 630°C. This weight loss was due to the combination of graphene in air. SnO_2_ inside the composite matrix could withstand up to 1000°C temp. Thus in SnO_2_/G nanocomposites, 35.2% weight is for SnO_2_ and 64.8% is for graphene.

**Fig 5:**
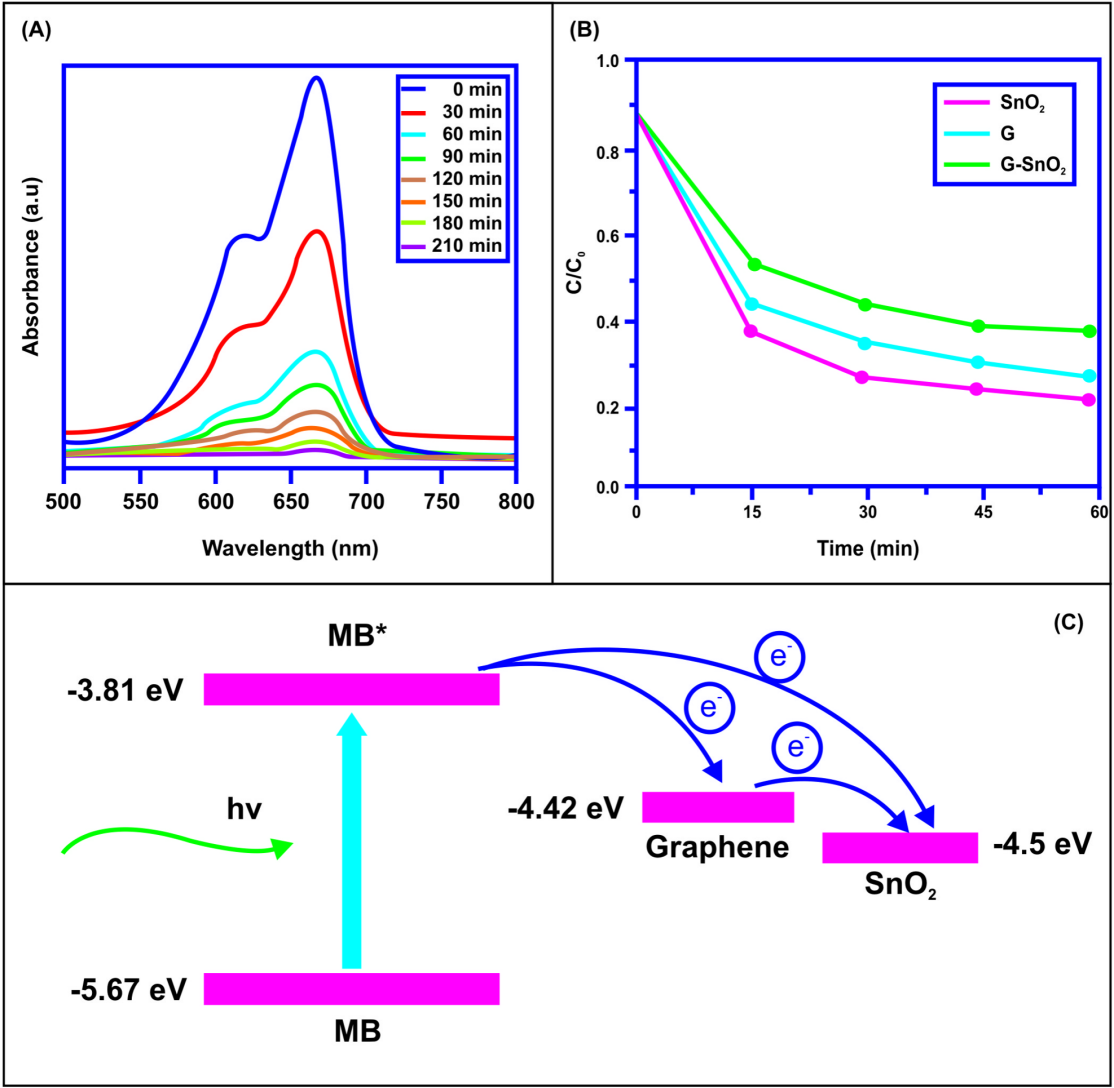
(a) Typical UV–vis absorption spectra of MB solutions after a certain time of UV irradiation in the presence of (a) SnO_2_/G (b) Photocatalytic degradation efficiency of the MB solution of SnO_2_, G and SnO_2_/G (c) The illustration of electron transfer among MB, graphene sheets and SnO_2_ nanoparticles.

Under visible light irradiation, the photocatalytic activity of the prepared samples was evaluated using methylene orange (MO) dye. (Fig 5A and 5B) illustrates the UV-Vis absorption spectra of the SnO_2_/G nanocomposite with adsorbed MO for different time ranges. However, for SnO_2_/G nanocomposite showed 100% degradation efficiency under visible light irradiation for the time duration of 6h (Fig 5(A)). The adsorption of MO takes place on the surface of the graphene as well as on the surface of the metal oxides using the synthesized composites. The homogeneous distribution of SnO_2_ nanoparticles over the surface of graphene sheets provides large surface area of the composite material. The enhanced surface area contributes to the improved photocatalytic activity of the SnO_2_/G nanocomposites. The C/C_0_ values were calculated using the equation, C/C_0_ = kt, where, C and C_0_ are the preliminary and calculated absorption of the MO solution over the time interval of t. k represented the photo-degradation time constant. The values for C/C_0_ versus the irradiation time were plotted and Fig 5(B) illustrates it. The synthesized composite showed better photo-degradation efficiency compared with pure SnO_2_ nanoparticles. The process of electron transfer at the interface of SnO_2_/RGO nanocomposite for photocatalytic degradation of MB has been explained by previous researchers [48, 49]. Fig 5(C) explains well the process of electron transfer for MB at normal condition (5.67eV) and excited (MB*) (3.81eV) condition including graphene (4.42eV) and conduction band of SnO_2_ (4.5eV). The adsorption of MB takes place both over the surface of SnO_2_ nanoparticles as well as over the surface of graphene. Under UV light irradiation MB is excited to give MB*. The electrons from MB* would get transferred to graphene and give positively charged MB* ions. The electrons over the graphene will recombine with MB*. Overall, it results in slow degradation of MB over the RGO [50]. The conduction band of SnO_2_ will collect the electrons due to its higher work function than graphene [Fig 5C]. The electrons will be presented to contact with MB* (hole). The dissolved oxygen inside the water would capture the electrons from the surface of the SnO_2_ nanoparticles. Consequently, it will give highly reactive oxygen species which would oxidize the MB molecules [51]. RGO would work as electron mediator. Presence of RGO would facilitate the electron transfer process from MB* to SnO_2_. Overall, this would result in higher photocatalytic efficiency of SnO_2_/RGO nanocomposites compared with bare SnO_2_ nanoparticles.

**Fig 6:**
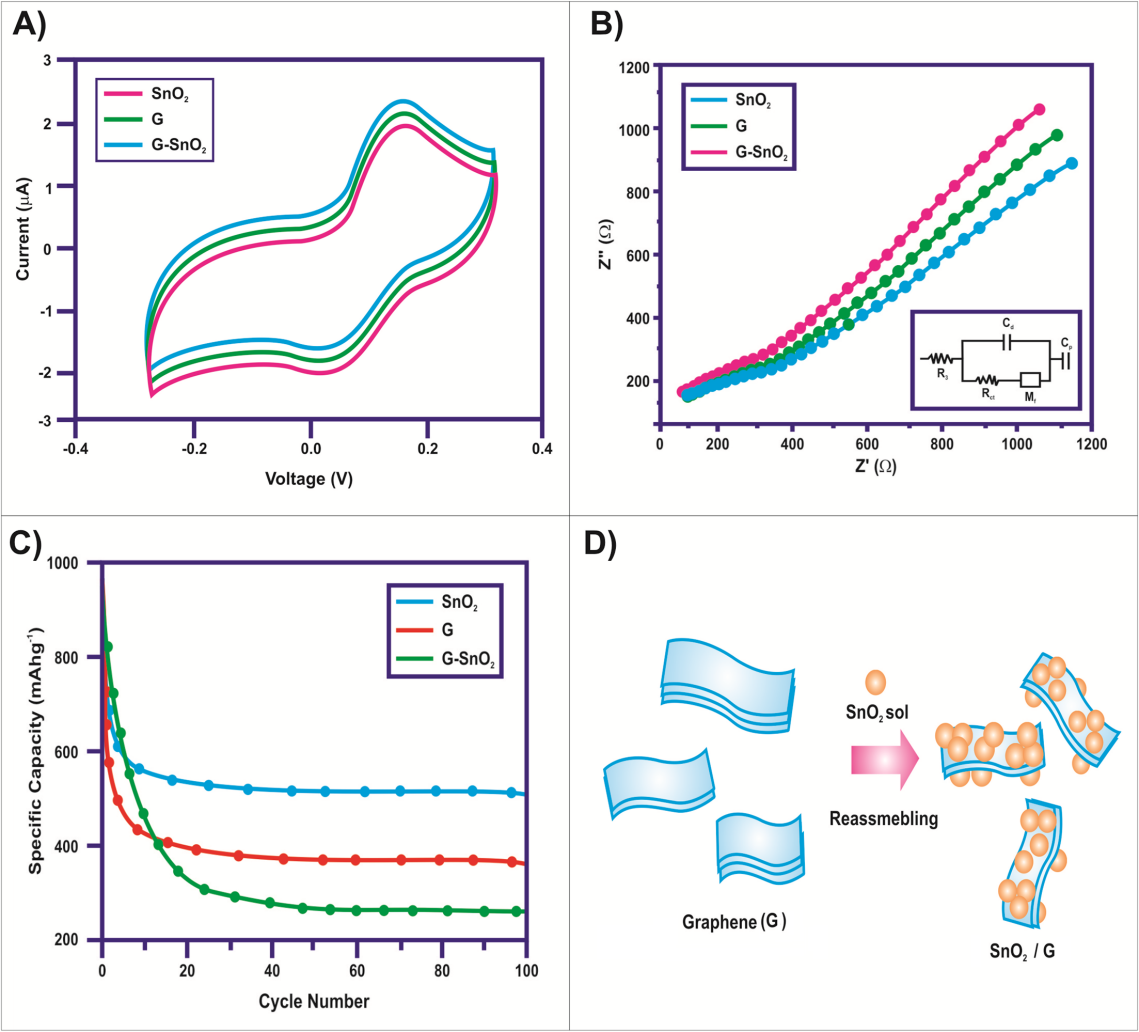
(a) Cyclic voltammetry response of SnO_2_/G nanocomposites (b) Nyquist Plots of SnO_2_/G-Nanocomposite (c) Cycling performances of SnO_2_/G nano composite (d) Schematic illustration of synthesis process and structure of SnO_2_/G [57].

The cyclic voltammetry process was carried out to observe the electrochemical performance of the synthesized SnO_2_/G nanocomposites for supercapacitor. Cyclic voltammetry behavior of the synthesized sample is illustrated in Fig 6(A). From the figure, it could be observed that all the CV curves for the electrodes are symmetrical in nature. This is basically characteristic curve showing ideal capacitive behavior with quasi rectangular shape. This indicates excellent capacitive behavior including low contact resistance of the super capacitor electrodes. [52]. Different scan rates obtained for the potential range of -0.4 to 1 V, did not exhibit any broad redox peaks. This showed that the electrodes were consecutively charged and discharged over the whole voltammetry cycle [53, 54]. The CV curves obtained for the composite electrodes were more rectangular and showed relative increment of current level compared with pure SnO_2_ sample. Thus the overall specific capacitance for the synthesized sample was due to the EDLC and pseudo-capacitance behavior of the composite materials. The capacitance obtained for pure SnO_2_ electrodes was only for pseudo-capacitance. The charge transfer process is illustrated by semi-circular arc. The inclined lines represent the irregular diffusion of charged species towards the modified electrode. When the interfacial charge transfer resistance is high; it gives large semicircular curves. This results from the poor electrical conductivity of the active substances. If the electrode characteristics are closer to the ideal capacitor, then at lower frequency level more vertical lines are observed. The process taking place at the electrode/electrolyte interfaces at higher frequency level showed the depressed arc (Fig 6(A)). The solution resistance (Rs) at higher frequency level could be observed from the intersections of curves at real axis [55]. Basically the solution resistance (Rs) shows the ionic conductivity of electrolyte solution. The absence of semicircular curves at the higher frequency level of the electrodes is due to low Faradic charge transfer resistances [56].

Fig 6(B) shows the Nyquist plots of SnO_2_ and SnO_2_/G nanocomposite. The synthesized composite showed higher conductivity than the pure SnO_2_ sample. Thus the diameter of the semicircle obtained for the composite was smaller than for the pure SnO_2_ sample at higher frequency level. The higher frequency region for the impedance plot and the fitting circuit for SnO_2_/G are shown by the inset figures. Rs and Rct represent the solution and charge transfer resistances respectively. The solution resistance (R) of the cell is calculated from the intercept between the impedance plot and the real impedance of (Z’) axis. The charge transfer resistance (R) of the interface between the electrode and electrolyte is calculated from the diameter of the semi-circle. The ionic and electronic resistances as well as the intrinsic resistance of the modified electrodes and the diffusive and contact resistances at the composite modified electrodes will combine together to give solution resistances. The electrochemical reaction on the electrode/electrolyte interface is more simplistic for SnO_2_/G and smaller than for the pure SnO_2_ sample resulting in smaller values of Rct for the composite electrodes. The magnitude of the EIS values also clearly indicates the better capacitive behavior of the composite electrode than the pure one.

Fig 6(C) shows the long-term cycling properties of the SnO_2_/G nanocomposite, bare graphene nanosheets and SnO_2_ nanoparticles at a 0.1 C current rate. The highest reversible capacities with the best cycling stability were observed for the SnO_2_/G composite electrode. The nano composite electrode delivered a discharge capacity in the first cycle and maintained stable capacities from the second cycle for 100 cycles with an excellent capacity retention. Compared with the composite electrode, graphene electrode showed larger irreversible capacity with lower discharge capacities for 100 cycles. The capacities of SnO_2_ nanoparticles decreased quickly upon cycling. In the experiments, reduced graphene nanosheets and the as-prepared SnO_2_ nanoparticles were reassembled in ethylene glycol solution to construct a 3-D flexible structure (Fig 6(D)) [57].

## 4.Conclusions

SnO2/Graphene nanocomposites were synthesized successfully via a facile hydrothermal method. The crystalline phase of SnO_2_ nanoparticles was observed by XRD analysis. HR-TER images confirmed the presence of SnO_2_ nanoparticles over the surface of graphene sheets. The uniform distribution of SnO_2_ nanoparticles was observed over the wrinkled graphene nanosheets. The morphological analysis using FE-SEM studies showed that the pure graphene had a wrinkled paper like structure. However, the SnO_2_/G nanocomposite showed the island like structure where the SnO_2_ nanoparticles were coated over the graphene sheets. The synthesized sample showed a red shift in the absorption spectra. The FT-IR results confirmed the functional groups of graphene and SnO_2_ nanoparticles. Most of the bands containing C = O and C-O functional groups had disappeared from SnO_2_/G nanocomposites. The presence of G and D band peaks as well as the metal oxide peaks was confirmed by the Raman analysis. The TGA results showed that SnO_2_/G possessed higher thermal stability. The enhanced photocatalytic performance for the degradation of MO and MB dyes under the visible and UV light for the synthesized sample reflected its potential application for industrial waste water treatment. The electrochemical performance of the synthesized sample was enhanced due to the presence of graphene inside its matrix. The desalination performance for the composite was greatly increased due to excellent adsorption properties and electrical conductivity of the graphene nano sheets. The surface area of the graphene sheets was greatly reduced by its aggregation intensities. Incorporation of SnO_2_ inside the graphene layers with prevents its aggregation. The amount of SnO_2_ needs to be carefully optimized to prevent self-agglomeration of SnO_2_ nanoparticles. This could be attributed to its enhanced specific capacitance, better stability as well as increased wettability of the electrode materials.

## Supporting information

S1 Text (DOC)
