# Supplementary material for: A facile hydrothermal approach for catalytic and optical behavior of tin oxide- graphene (SnO2/G) nanocomposite
Source: PLoS One. 2018 Oct 1;13(10):e0202694. doi: 10.1371/journal.pone.0202694 (PMC6166922; doi:10.1371/journal.pone.0202694)
Supplement: S1 Text — (DOC) [file pone.0202694.s001.doc]

**Supporting Information: S1**

The working electrode was prepared by mixing the electro active material of (a) SnO_2_/G (80 wt %), (b) activated carbon (10 wt %), and (c) polyvinylidene fluoride (10 wt %). Finally, the acquired slurry was coated onto (d) nickel foil (1 cm^2^) and dried at 100 °C in air for 12 h. Every electrode included nearly 1 mg of electro active material. The SnO_2_/G electrode as the working electrode, Pt wire as counter electrode and the saturated calomel electrode as reference were employed for further study. Aqueous 2 M KOH solution was used as the supporting electrolyte. CVs in the potential range of -0.4 to 1 V at various current densities and electrochemical impedance spectra (EIS) of amplitude were carried out by using VSP biologic electrochemical system.
